# Well-being, gender, and psychological health in school-aged children

**DOI:** 10.1186/s13690-015-0104-x

**Published:** 2015-12-21

**Authors:** Isabelle Savoye, Nathalie Moreau, Marie-Christine Brault, Alain Levêque, Isabelle Godin

**Affiliations:** Service d’Information Promotion Education Santé (SIPES), School of Public Health, Université Libre de Bruxelles (ULB), Bruxelles, Belgium; Research Centre in Epidemiology, Biostatistics and Clinical Research, School of Public Health, Université Libre de Bruxelles (ULB), Bruxelles, Belgium; Institut de recherche en santé publique, Université de Montréal, Montréal, Canada

**Keywords:** Gender differences, Psychological health complaints, Adolescence, Well-being

## Abstract

**Background:**

Despite being a well-documented phenomenon, gender differences in psychological health complaints in adolescence are poorly understood. The purpose of this study was to test factors related to well-being as explanatory factors of gender differences in psychological complaints (feeling low, irritability or bad temper, nervousness, and sleeping difficulties) in adolescence.

**Methods:**

This study was based on the 9^th^ Health Behaviour in School-aged Children (HBSC) study, conducted in 2010 in the Wallonia-Brussels Federation, Belgium, on 9–24 year olds. Using logistic regression analyses, we studied gender differences in psychological complaints through well-being factors (life satisfaction, self-confidence, helplessness, and body image), across age categories, and examined the variation of female excess after taking into account each factor.

**Results:**

The four well-being factors together explained more than half of the female excess in feeling low. However, there were still significant gender differences in feeling low for children over 13. Among 13 to 15-year-olds, there were no gender differences in irritability after adjustment. An important decrease in gender differences in nervousness was observed in the multivariate analyses, although there was still significant female excess in nervousness increasing from 13 years old. After full adjustment, only gender differences in sleeping difficulties among 13–15-year-olds remained significant. For all psychological complaints studied, self-confidence caused the most important decrease in gender difference.

**Conclusions:**

This study showed that factors related to well-being could mediate the association between gender and psychological complaints, and pointed to the importance of taking into account well-being factors in the analyses of the aetiology of gender differences in psychological complaints. However, our results suggested that future research should explore additional explanations for gender differences in psychological complaints.

## Background

Many studies have shown that subjective health complaints are more prevalent among adolescent girls than boys [1–9] and that gender differences among adolescents intensify with age [2, 3, 6, 7, 10, 11]. For instance, several studies have found higher levels of subjective health complaints in 15-year-old girls among 11–15-year-old adolescent populations [2, 6, 9].

Although subjective health complaints do not always correspond to a specific diagnosis, they provide some discomfort, impact the quality of life, and have been related to global well-being [4]. They are characterized by two dimensions: somatic (headaches, backaches) and psychological (nervousness, irritability) that could have different aetiologies [3, 10]. Macintyre et al. have found that the direction and the magnitude of sex differences in health varied according to particular symptoms and phases of the life cycle [12]; a female excess was not always apparent and sometimes reversed for a number of physical symptoms, but appeared to be consistently found across the lifetime for psychological symptoms. In this study, we will thus focus on the psychological facet of subjective health complaints included in the Health Behaviour in School-aged Children (HBSC) study, which includes feeling low, irritability or bad temper, nervousness, and sleeping difficulties.

Sweeting et al. have suggested that the excess of subjective health complaints reported by adolescent girls could be partly explained by a lower level of psychological well-being during adolescence [13]. Indeed, girls are disadvantaged on measures related to well-being, such as self-confidence, self-esteem, happiness, and powerlessness compared to boys [4]. These well-being factors have also been associated with subjective health complaints such as sleeping difficulties, tiredness, sore muscles, etc. among adolescents [4, 14]. Therefore, the way that adolescents perceive themselves could explain the majority of gender differences in depressive symptoms [15]. Most research has studied the way in which various factors (demographic, family, school, peers, behaviours, etc.) influence health disorders separately for boys and girls [1, 6, 14, 16–18]. But only a few studies have attempted to identify which factors related to well-being influence gender differences in psychological health symptoms.

In 2003, Rutter et al. [19] reviewed the research strategies that may be employed to study the causal mechanisms underlying sex differences in psychopathology. They suggested that a mediation model could be of interest. Rather than being direct, the association between gender and psychopathology could be indirect through a potential explanatory factor, also called a mediator: gender would be associated with the mediator which in turn would be associated with the outcome (psychopathology). This means that the adjustment for this potential explanatory factor (the mediator) could contribute to the explanation of the gender differences in the health outcome.

This is also the method used by Sweeting et al. when studying female excess in psychosomatic symptoms in adolescence. In this study, Sweeting et al. [20] have studied the contribution of a range of factors to female excess in headaches, stomach ache, dizziness, and depressed mood. Interestingly, they showed that adjusting for well-being (self-esteem, body image) and behavioural (current smoking, physical activity) factors largely explained the female excess in depressive mood. However, depressive mood was the only psychological component studied, and the analyses only included pupils aged 15 years old, which does not permit an examination of differences across the age range.

Other studies also indicated that gender differences in depression were eliminated after controlling for psychosocial variables [21] or early adolescent challenges [22]. Takakura and Sakihara found that gender differences in depressive symptoms were eliminated after controlling for psychological variables such as life stresses, social support, health practices, self-esteem, and locus of control [23], suggesting that if girls had the same levels of these psychological factors as did boys, they would not report so many depressive symptoms.

The purpose of this study is to test four factors related to well-being (life satisfaction, self-confidence, helplessness, and body image) as explanatory factors of gender differences in psychological complaints (feeling low, irritability or bad temper, nervousness, and sleeping difficulties), among different age categories.

## Methods

### Study design

The HBSC study is a cross-national survey supervised by the WHO Regional Office for Europe, including countries and regions across Europe and North America. The survey collects data every four years on students’ health and well-being, social environments, and health behaviours. The HBSC study took place in the Wallonia-Brussels Federation (FWB) in 1986 for the first time, and represents the French-speaking side of the Belgian part of the international study. The present study is based on the 9^th^ survey, conducted in 2010 in FWB by the SIPES Service (Service d’Information Prévention Education Santé) among students from the 5^th^ grade (elementary) to the last grade (secondary), which includes pupils from 9 to 24 years old.

The HBSC survey was conducted on a random sample stratified proportionally to the distribution by province and by school network of the school population. It is a two-step cluster design, with, first, a school selection, and second, a class selection. The sample is representative of schoolchildren from the FWB. Children responded anonymously to the questionnaire during class-time under teacher supervision.

In FWB, before the start of the investigation, the school networks authorities must provide us their approval to contact the schools. To do so, the school networks authorities have access to the questionnaires to be completed by the students and, if necessary, some questions may be edited or deleted. When the approval is obtained from the schools networks, schools are invited to participate to the study, but are free to participate or not. In 2010, the participation rate from the schools that responded to the request to participate was around 50 %. Overall, children and youths from 157 primary classrooms (5^th^ and 6^th^ grade) and 513 secondary classrooms were interviewed. Of 10,710 completed questionnaires, 10,533 had available information on sex and age and could be retained for analysis. Students aged over 22 years (*n* = 17) were excluded from the analyses, leading to a final sample of 10,516 pupils. More details about the methodology (e.g. sampling, data collection) are available in the FWB reports [24–26].

### Measures

The HBSC questionnaire was firstly elaborated in English and then translated into the respective languages by the national or regional teams. A back translation into English was performed by an independent translator and validated by the international coordinator to ensure the comparability between countries and regions.

The HBSC survey asks youth to report on four psychological health complaints: feeling low, irritability or bad temper, nervousness and sleeping difficulties. The questions ask how frequently during the last 6 months the symptom occurred (*rarely or never, about every month, about every week, more than once a week, about every day*). Following similar categorisation in many international reports and studies [2, 4, 9, 27], each psychological health complaint was dichotomised, and considered as recurrent if reported more than once a week (*about every day*, *more than once a week* vs. *about every week*, *about every month*, *rarely or never*).

The well-being related factors included in this study were life satisfaction, self-confidence, helplessness, and body image. Life satisfaction is an evaluation of the quality of life and an important aspect of well-being [28]. It was obtained with the Cantril ladder that asks the child to quote his life satisfaction on a scale from 0 to 10 (0 being the worst possible life and 10 the best possible life). As in other studies [2, 9, 24, 29], the variable was dichotomised according to *low* (0–5) and *normal-high* (6–10) life satisfaction. Self-confidence and helplessness were measured by the following questions: ‘How often do you feel confident about yourself?’ and ‘How often do you feel helpless?’ Responses were dichotomised: *always/ often* and *sometimes/rarely/never* [24, 25]. Finally, the body image was assessed by asking children how they perceive their bodies. Response options were categorised as follows: *much/a bit too fat* and *much/a bit too thin/alright* [2, 9].

In order to assess developmental patterns of gender differences in psychological complaints, we categorised age into four groups: 9 to 12 years old (end of elementary school), 13 to 15 years old (beginning of high-school/puberty), 16 to 18 years old (mid/late adolescence), and 19 to 22 years old (end of adolescence/beginning of adulthood).

### Statistical analysis

To explain gender differences in psychological complaints through well-being factors, we followed the three steps recommended by Rutter et al. [19] to consider a factor as a mediator: the first step aims to test the association between gender and the factor, the second step is to test the association between the factor and the health measure, and the last step tests for the association between gender and the health measures including the factor within the analysis. If the factor is associated with both gender and the health outcome, it could mediate the gender difference in that health outcome. This means that the adjustment for this potential explanatory factor could eliminate or reduce the association between gender and the health outcome.

Therefore, we hypothesised that the well-being factors were associated with gender on the one hand and with psychological complaints on the other hand. Well-being factors among girls were compared to those among boys using Pearson chi-squared tests. Logistic regression analyses were performed to estimate odds ratios (OR) and their 95 % confidence intervals of reporting each psychological health complaint more than once a week for well-being factors.

To study gender differences in psychological complaints through well-being factors, logistic regression analyses were performed. First, odds ratios and their 95 % confidence intervals of the female excess for each psychological health complaint were obtained from univariate logistic regression analyses. Odds ratios of the female excess were then calculated after adjusting for each potential explanatory factor separately. Finally, multivariate models were produced, which were adjusted for all factors together.

To better appreciate the indirect effect of gender on psychological complaints through well-being factors, the variation of the female excess after taking into account each factor/all factors was calculated, representing the proportion of the gender difference explained by the/all factor(s). The proportion of the gender difference variation in psychological health complaints explained by the well-being factor was calculated as follows: [1-[(OR_ajusted_-1)/(OR_univariate_-1)]]*100 [20].

Because of the well-known interaction between gender and age [2, 3, 6, 7, 10, 11], all analyses were performed separately for age-groups. The Hosmer-Lemeshow test for goodness of fit was performed for all logistic regression models and potential problems of collinearity were checked. All analyses were performed using STATA version 12 (College Station, TX: StataCorp LP).

## Results

Among the 10 516 questionnaires retained for study, there were 1 538 girls and 1 490 boys aged from 9 to 12 years old, 1 808 girls and 1 762 boys aged from 13 to 15 years old, 1 685 girls and 1 480 boys aged from 16 to 18 years old, and 353 girls and 400 boys aged from 19 to 22 years old. The proportion of adolescent girls reporting feeling low or having sleeping difficulties was significantly higher compared to boys, for all age categories (Table 1). Significant gender differences appeared from 13 to 15 years old for irritability and nervousness.

**Table 1:** Description of boys and girls reporting each psychological complaint by age (Chi-squared test)

	9–12					13–15					16–18					19–22				
Psychological complaint	Boys		Girls		*p*	Boys		Girls		*p*	Boys		Girls		*p*	Boys		Girls		*p*
	*n*	%	*n*	%		*n*	%	*n*	%		*n*	%	*n*	%		*n*	%	*n*	%	
Feeling low	1414	100	1474	100		1625	100	1728	100		1391	100	1636	100		374	100	346	100	
>1/week	130	9.2	196	13.3	***	188	11.6	362	21.0	***	176	12.7	384	23.5	***	48	12.8	83	24.0	***
Irritability/bad temper	1411	100	1464	100		1628	100	1729	100		1385	100	1636	100		378	100	343	100	
>1/week	215	15.2	249	17.0	NS	305	18.7	450	26.0	***	287	20.7	494	30.2	***	81	21.4	116	33.8	***
Nervousness	1408	100	1457	100		1619	100	1723	100		1400	100	1637	100		378	100	343	100	
>1/week	256	18.2	276	18.9	NS	332	20.5	523	30.4	***	334	23.9	579	35.4	***	97	25.7	154	44.9	***
Sleeping difficulties	1419	100	1475	100		1630	100	1734	100		1392	100	1647	100		378	100	344	100	
>1/week	405	28.5	495	33.6	**	406	24.9	622	35.9	***	364	26.2	531	32.2	***	116	30.7	130	37,8	*

***: *p* <0.001, **: *p* <0.01, *: *p* <0.05, NS: non significant

Following the three steps recommended by Rutter et al. [19], the distribution of the well-being factors by gender and the associations between well-being factors and psychological health symptoms were studied across age groups. Our results showed a significant disadvantage for girls compared to boys in life satisfaction, self-confidence, helplessness, and body image at all ages, and showed significant associations between each well-being factor and psychological health symptoms (except for body image on feeling low and sleeping difficulties and for helplessness on sleeping difficulties among the 19 to 22-year-old pupils) (data not shown). This suggested that well-being factors constitute potential explanatory factors that could explain partially or totally the association between gender and psychological complaints. Therefore, their impact on gender differences in psychological complaints was studied in logistic regression analyses.

Table 2 shows the odds ratios of the female excess for each psychological complaint using univariate and multivariate analyses after adjusting for potential explanatory factors, separately and together. The proportions of the OR decrease, after adjustment, are also presented.

**Table 2:** Regression analysis: odds ratios of female excess for health measures

	9–12		13–15		16–18		19–22	
	OR (95 % CI)	%^e^	OR (95 % CI)	%^e^	OR (95 % CI)	%^e^	OR (95 % CI)	%^e^
FEELING LOW								
Gender (girls vs. boys)	1.51 (1.20–1.82)		2.03 (1.67–2.45)		2.12 (1.74–2.57)		2.14 (1.45–3.17)	
Adjusted for:								
Life satisfaction^a^	1.43 (1.11–1.83)	16 %	1.86 (1.51–2.29)	17 %	2.06 (1.67–2.54)	5 %	2.12 (1.38–3.26)	2 %
Self-confidence^b^	1.27 (0.99–1.61)	47 %	1.51 (1.24–1.85)	50 %	1.42 (1.15–1.75)	63 %	1.69 (1.12–2.53)	39 %
Helplessness^c^	1.35 (1.07–1.72)	31 %	1.70 (1.39–2.07)	32 %	1.75 (1.43–2.14)	33 %	1.59 (1.05–2.40)	48 %
Body image^d^	1.46 (1.16–1.86)	10 %	1.90 (1.56–2.31)	13 %	1.85 (1.51–2.26)	24 %	2.11 (1.41–3.15)	3 %
All 4 well-being-related factors	1.20 (0.93–1.56)	61 %	1.42 (1.14–1.78)	59 %	1.32 (1.05–1.66)	71 %	1.66 (1.05–2.63)	42 %
IRRITABILITY/BAD TEMPER								
Gender (girls vs. boys)	1.14 (0.93–1.39)		1.53 (1.29–1.80)		1.65 (1.40–1.96)		1.87 (1.34–2.61)	
Adjusted for:								
Life satisfaction	1.05 (0.85–1.30)	/	1.40 (1.18–1.66)	25 %	1.59 (1.33–1.89)	9 %	1.85 (1.31–2.60)	3 %
Self-confidence	0.94 (0.76–1.16)	/	1.25 (1.05–1.48)	53 %	1.35 (1.13–1.62)	46 %	1.61 (1.14–2.27)	30 %
Helplessness	1.02 (0.83–1.26)	/	1.34 (1.13–1.59)	36 %	1.43 (1.20–1.70)	34 %	1.63 (1.15–2.30)	28 %
Body image	1.10 (0.90–1.34)	/	1.41 (1.19–1.67)	23 %	1.52 (1.28–1.81)	20 %	1.75 (1.25–2.47)	14 %
All 4 well-being-related factors	0.88 (0.71–1.10)	/	1.13 (0.94–1.36)	75 %	1.24 (1.03–1.50)	63 %	1.51 (1.05–2.17)	41 %
NERVOUSNESS								
Gender (girls vs. boys)	1.05 (0.87–1.27)		1.69 (1.44–1.98)		1.75 (1.49–2.05)		2.36 (1.72–3.23)	
Adjusted for:								
Life satisfaction	1.00 (0.82–1.22)	/	1.52 (1.29–1.80)	25 %	1.67 (1.41–1.96)	11 %	2.38 (1.72–3.28)	/
Self-confidence	0.90 (0.74–1.09)	/	1.38 (1.17–1.63)	45 %	1.46 (1.23–1.72)	39 %	2.00 (1.45–2.77)	26 %
Helplessness	0.96 (0.79–1.17)	/	1.48 (1.26–1.74)	30 %	1.57 (1.33–1.75)	24 %	2.08 (1.51–2.88)	21 %
Body image	1.03 (0.85–1.24)	/	1.60 (1.36–1.88)	13 %	1.68 (1.42–1.98)	9 %	2.26 (1.64–3.12)	7 %
All 4 well-being-related factors	0.85 (0.69–1.04)	/	1.24 (1.04–1.48)	65 %	1.40 (1.17–1.67)	47 %	1.98 (1.40–2.78)	28 %
SLEEPING DIFFICULTIES								
Gender (girls vs. boys)	1.26 (1.08–1.48)		1.69 (1.45–1.96)		1.34 (1.15–1.57)		1.37 (1.01–1.87)	
Adjusted for:								
Life satisfaction	1.22 (1.04–1.44)	15 %	1.56 (1.34–1.82)	19 %	1.32 (1.12–1.56)	6 %	1.34 (0.98–1.84)	8 %
Self-confidence	1.13 (0.96–1.33)	50 %	1.44 (1.24–1.69)	36 %	1.13 (0.95–1.34)	62 %	1.21 (0.88–1.67)	43 %
Helplessness	1.19 (1.02–1.40)	27 %	1.52 (1.30–1.77)	25 %	1.24 (1.05–1.45)	29 %	1.31 (0.95–1.79)	16 %
Body image	1.24 (1.06–1.46)	8 %	1.58 (1.36–1.84)	16 %	1.26 (1.07–1.49)	24 %	1.35 (0.99–1.86)	5 %
All 4 well-being-related factors	1.11 (0.93–1.31)	58 %	1.32 (1.12–1.55)	54 %	1.12 (0.93–1.33)	65 %	1.26 (0.90–1.77)	30 %

^a^Low vs. Normal/High

^b^Sometimes/rarely/never vs. Always/often

^c^Always/often vs. Sometimes/rarely/never

^d^Much/a bit too fat vs. Much/a bit too thin/alright

^e^Proportion of the gender difference in psychological complaints explained by the factor

/The proportion was not calculated since gender difference in that psychological complaint was not significant in univariate analyses

The results showed a decrease in gender differences in feeling low after adjusting for each well-being factor separately. Among those, lack of self-confidence (up to 63 % in 16–18-year-old pupils) and helplessness (up to 48 % in 19–22-year-old pupils) explained most of the decrease in gender difference. After all well-being factors were controlled for, the gender difference in feeling low decreased more than 50 %, except for the 19–22-year-olds (42 %), meaning that those four factors explained together more than half of the female excess in this psychological symptom. However, gender differences in feeling low remained significant from 13 years old.

Self-confidence also caused the most important decrease in gender differences in irritability. Among the 13–15-year-olds, there was no longer a gender difference in irritability after adjustment for all well-being factors, which explained 75 % of the gender difference in irritability. The gender difference decrease was about 63 % in the 16–18-year-olds and 41 % in the 19–12-year-olds, and was at the limit of significance after the adjustment.

The impact of these well-being factors was somewhat similar for gender differences in nervousness although a little less important than for irritability. In all age groups, an OR decrease was observed after controlling for the four well-being factors; the greatest decrease was for the 13–15-year-olds (65 %), although there was still a significant female excess in nervousness in this age group. As with feeling low and irritability, it was self-confidence that had the major impact on gender difference (up to 45 % in 13–15-year-olds).

Well-being factors explained a major part of the female excess in sleeping difficulties. Again, lack of self-confidence causes most of the decrease in gender difference (up to 62 % in 16–18-year-old pupils). While there was a significant female excess in sleeping difficulties among all ages in univariate analyses, gender differences in sleeping difficulties remained significant only among 13–15-year-olds after adjustment for all well-being factors. Altogether, well-being factors explained more than 50 % of the female excess in sleeping difficulties (58 % for 9–12, 54 % for 13–15, and 65 % for 16–18), except for 19–22-year-olds (30 %).

For the 4 psychological health complaints studied, self-confidence was the factor that had the greatest influence on gender differences.

The variation of the female excess after adjusting for factors related to well-being permitted a better appreciation of their impact on gender differences in psychological health complaints across ages (Table 2). For feeling low, the impact of adjusting for well-being factors on gender differences seemed to increase from the 9–12-year-olds to the 16–18-year-olds and to be less important among the 19–22-year-olds. However, the impact of adjusting for well-being factors (separately or together) on gender differences in irritability and nervousness decreased across age categories, and there was no particular trend of the impact of adjusting for well-being factors on gender differences in sleeping difficulties. For all psychological complaints studied, the adjustment for well-being factors seemed to have a minor impact among the 19–22-year-olds compared to the other age groups.

## Discussion

The objective of this study was to test four factors related to well-being (life satisfaction, self-confidence, helplessness, and body image) as explanatory factors of gender differences in psychological complaints (feeling low, irritability or bad temper, nervousness, and sleeping difficulties), among different age categories.

In order to test for a mediation effect, we tested if well-being factors were associated with gender on the one hand and with psychological complaints on the other hand. The results showed that the four well-being factors (life satisfaction, self-confidence, helplessness, and body image) were associated with gender (with a disadvantage for girls) and with each psychological symptom; they therefore constitute potential explanatory factors that could mediate gender differences in psychological complaints.

The reduction (or elimination) of gender differences in psychological symptoms after controlling for these potential explanatory factors was therefore examined. Analyses showed a large reduction of gender differences in psychological complaints after the adjustment. After the well-being factors were controlled for, gender differences seemed to appear later in adolescence, in relation to feeling low and nervousness for the 13–15-year-olds and in relation to irritability for the 16–18-year-olds. A female excess in sleeping difficulties remained significant only for the 13–15-year-olds. Among the four well-being factors, self-confidence caused the greatest reduction of gender differences in psychological complaints. The impact of these well-being factors seemed to be more important among the 13–15 and the 16–18-year-olds.

### Importance of gender

Consistent with our findings, gender differences in some factors related to well-being, such as life satisfaction [29], self-confidence [17], or body image [30], with a disadvantage for girls have been reported by previous studies.

Furthermore, gender differences in psychological complaints are well-documented [1, 3, 6, 7, 20]. A cross-national study in Europe and North America showed different patterns leading to an increase of gender differences with age [7]. For low feeling, irritability, and nervousness, the increasing gender difference resulted from a strong increase in girls reporting these symptoms across age groups, while for sleeping difficulties the increasing gender difference was due to a decrease in this symptom for boys. This is consistent with our findings, except that our results showed no specific age-related pattern for sleeping difficulties.

### Association between well-being factors and psychological complaints

The relation between well-being factors and health has also been studied previously. Piko et al. examined potential protective factors operating in three contextual domains (parental, school-related, and individual) and revealed that individual level variables (i.e. life satisfaction and optimism) were important predictors of adolescent depressive symptomatology [31]. One study showed that self-confidence predicted good health for the next two years among boys [17]. The 2010 HBSC study in FWB showed that happiness was more frequent among young people who reported few health complaints [25].

### Gender differences in psychological complaints explained by well-being factors

Eiser et al. [14] have found that gender differences in physical and psychological symptom reporting strongly decreased after taking into account self-reported confidence, competence, family support, and social responsibility. Moreover, Sweeting et al. [20] observed an important reduction of gender differences in somatic complaints and depressive mood after controlling for psychosocial and behavioural factors. For depressive mood, self-esteem was the most influential factor and there were no more gender differences after all factors were controlled for. Even if our study had showed similar results, gender differences in psychological complaints remained significant after adjustment, suggesting that additional factors are needed to explain the female excess.

Other authors have also suggested that gender differences in depressive symptoms could be explained by the female excess on the psychological variables studied [23]. For example, Bennett et al. have observed that depressed girls experienced more sadness/depressed mood, body image dissatisfaction, sleeping difficulties, and health worries than depressed boys [32], and Danielsson et al. found that sleep disturbances, which were predictors of depressive symptoms one year later, were reported more by girls than boys [33]. Thus, since girls are disadvantaged in regards to factors related to well-being, also associated with psychological complaints, these associations could explain the association between gender and psychological complaints. In other words, the lower levels of life satisfaction, self-confidence, and higher levels of helplessness and body image dissatisfaction for adolescent girls could contribute to their greater psychological complaints.

While boys are less healthy than girls during childhood, the reverse is true during adolescence, suggesting that an important change connected with gender occurs during puberty [34]. As an example, Benjet and Hernandez-Guzman found no gender differences in depression, self-esteem, or body image among prepubertal youths. However, menarche led to an increase of these symptoms in girls, while there were no changes in depression for boys, who experienced an improvement in body image after their voices changed [34]. The explanation for the increasing female excess in psychological complaints with age could be that different experiences during puberty produce more physical and psychological changes in girls’ lives than in boys’ [34]. Thus, one theory suggests a non-negligible impact of gender-specific puberty experiences [1], like menstruation, that are associated with some physical and psychological symptoms for most girls (such as weight gain and body image dissatisfaction), but that are perceived in a positive way for boys [35]. This could explain why, in our study, well-being factors explained a larger part of the gender difference in psychological complaints among the 13–15 and 16–18-year-olds than among the 19–22-year-olds.

Our study has several strengths and weaknesses. The good representativeness and the sample size are some of strengths of this study, as is the use of four health measures to reflect the psychological dimension of health [3]. Although this study focused on the impact of factors related to well-being, other factors have been related to psychological complaints. In addition to measures of well-being, measures related to demographic characteristics, psychosocial status, and puberty should be considered in future analyses. As HBSC is a transversal survey, there is no possibility of evidencing a causal link between the associations found, such as between well-being factors and psychological complaints. Longitudinal studies are necessary to better understand the relationship between the factors studied and the psychological symptoms, but also to understand the effect of age from a developmental perspective.

## Conclusions

This study showed that factors related to well-being could mediate gender differences in psychological symptoms, and pointed to the importance of taking into account well-being factors in the analyses of the aetiology of gender differences in psychological health symptoms. However, our results suggested that future research should explore additional explanations for gender differences in psychological symptoms. These are necessary to more fully understand the origins of these differences.

## Abbreviations

95 % CI: 95 % Confidence Interval
HBSC: Health Behaviour in School-aged Children
FWB: Wallonia-Brussels Federation
OR: Odds Ratio
